# Postdevelopment Performance and Validation of the Artificial Intelligence-Enhanced Electrocardiogram for Detection of Cardiac Amyloidosis

**DOI:** 10.1016/j.jacadv.2023.100612

**Published:** 2023-09-14

**Authors:** David M. Harmon, Kathryn Mangold, Abraham Baez Suarez, Christopher G. Scott, Dennis H. Murphree, Awais Malik, Zachi I. Attia, Francisco Lopez-Jimenez, Paul A. Friedman, Angela Dispenzieri, Martha Grogan

**Affiliations:** a Department of Internal Medicine, Mayo Clinic School of Graduate Medical Education, Rochester, Minnesota, USA; b Department of Cardiovascular Medicine, Mayo Clinic College of Medicine, Rochester, Minnesota, USA; c Department of Quantitative Health Sciences, Mayo Clinic, Rochester, Minnesota, USA; d Department of Artificial Intelligence and Informatics, Mayo Clinic, Rochester, Minnesota, USA; e Division of Hematology, Mayo Clinic College of Medicine, Rochester, Minnesota, USA; f Department of Laboratory Medicine and Pathology, Mayo Clinic, Rochester, Minnesota, USA

**Keywords:** amyloidosis, arrhythmias, artificial intelligence, cardiomyopathy, electrocardiogram, heart failure, neural network

## Abstract

**Background:**

We have previously applied artificial intelligence (AI) to an electrocardiogram (ECG) to detect cardiac amyloidosis (CA).

**Objectives:**

In this validation study, the authors observe the postdevelopment performance of the AI-enhanced ECG to detect CA with respect to multiple potential confounders.

**Methods:**

Amyloid patients diagnosed after algorithm development (June 2019-January 2022) with a 12-lead ECG were identified (n = 440) and were required to have CA. A 15:1 age- and sex-matched control group was identified (n = 6,600). Area under the receiver operating characteristic (AUC) was determined for the cohort and subgroups.

**Results:**

The average age was 70.4 ± 10.3 years, 25.0% were female, and most patients were White (91.3%). In this validation, the AI-ECG for amyloidosis had an AUC of 0.84 (95% CI: 0.82-0.86) for the overall cohort and between amyloid subtypes, which is a slight decrease from the original study (AUC 0.91). White, Black, and patients of “other” races had similar algorithm performance (AUC >0.81) with a decreased performance for Hispanic patients (AUC 0.66). Algorithm performance shift over time was not observed. Low ECG voltage and infarct pattern exhibited high AUC (>0.90), while left ventricular hypertrophy and left bundle branch block demonstrated lesser performance (AUC 0.75 and 0.76, respectively).

**Conclusions:**

The AI-ECG for the detection of CA maintained an overall strong performance with respect to patient age, sex, race, and amyloid subtype. Lower performance was noted in left bundle branch block, left ventricular hypertrophy, and ethnically diverse populations emphasizing the need for subgroup-specific validation efforts.

Amyloidosis is a sinister condition with an often subtle, heterogenous presentation, frequently evading prompt diagnosis despite multiple specialist evaluations.^1^^,^^2^ While previously considered rare, recent studies suggest the disease has a higher prevalence than expected,^3^^,^^4^ with significant implications for disease identification and management.^2^^,^^5^ With the increased development of effective medical therapies,^6^, ^7^, ^8^, ^9^ there is a critical need for screening tools to appropriately heighten clinical suspicion and establish an early clinical diagnosis of amyloidosis prior to severe disease manifestation.^2^^,^^10^

Patients with early symptoms of systemic disease are frequently referred for multispecialty assessment. This often includes a cardiac workup with electrocardiogram (ECG), especially for those experiencing palpitations, dyspnea, or other cardiac symptomology. While cardiac imaging has improved the ability to identify amyloidosis, this disease entity frequently remains undiagnosed or unrecognized.^2^ As a result, our team applied artificial intelligence (AI) to standard 12-lead ECGs routinely obtained in clinical practice and validated a screening tool to detect and predict cardiac amyloidosis with good performance (area under the receiver operating characteristic [AUC] 0.91).^10^

Our work is similar to other AI-ECG networks developed at our institution to detect multiple cardiac pathologies from a 12-lead ECG (ie, aortic stenosis,^11^ heart failure,^12^ and hypertrophic cardiomyopathy^13^).^14^ While each of these AI models, including our own, has exhibited high accuracy and clinical promise, it remains critical to understand the strengths, limitations, and maintenance needs of each algorithm as they become increasingly accessible to practitioners in various clinical environments.^15^^,^^16^

The AI-ECG model for cardiac amyloidosis was initially developed and validated on a large, retrospective, well-defined cardiac amyloid patient cohort and analyzed with respect to multiple variables (ie, age, sex, and amyloid subtype) highlighting the potential of AI to identify this elusive pathology.^2^^,^^10^ This original investigation was also novel in applying the AI-ECG technology to single and 6-lead ECG systems as a proof-of-concept to apply this screening technology utilizing portable, mobile ECG tools.^10^ However, a knowledge gap remains regarding the algorithm’s performance postdevelopment with respect to specific sex/age groups, race/ethnicity, amyloid subtype, recording location, ECG features, and time after development. In this investigation, we discuss the performance of the algorithm with respect to these variables to better understand the performance and limitations of the AI-ECG model for cardiac amyloidosis (Central Illustration).

**Central Illustration:**
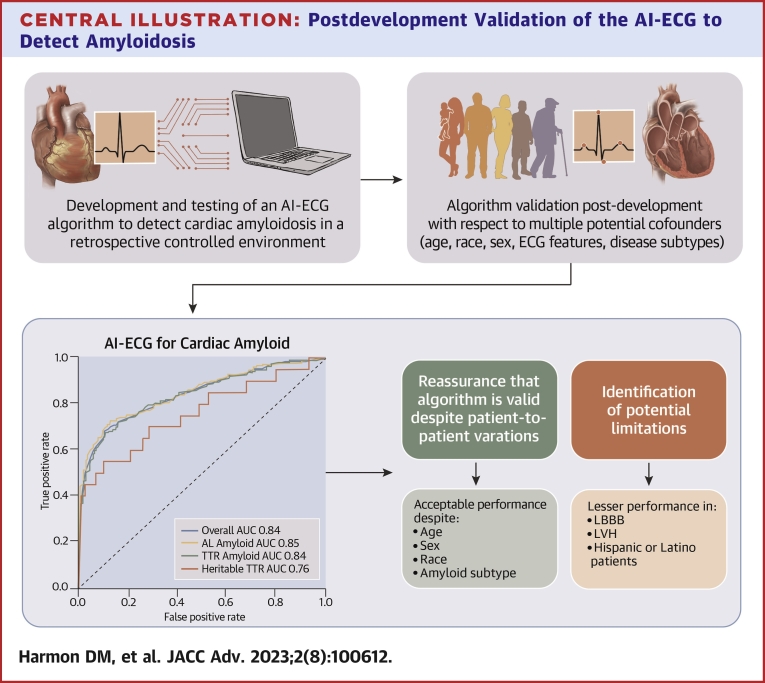
Postdevelopment Validation of the AI-ECG to Detect Amyloidosis After developing and testing an AI-ECG model to detect amyloidosis, it is important to validate the model postproduction to better understand the algorithm’s performance with respect to multiple potential confounders. This process allows for a deeper clinical understanding of the robustness and limitations of these AI health tools. The upper left image is from Grogan et al.^10^ AI = artificial intelligence; ECG = electrocardiogram.

## Methods

### Study population

**Figure 1:**
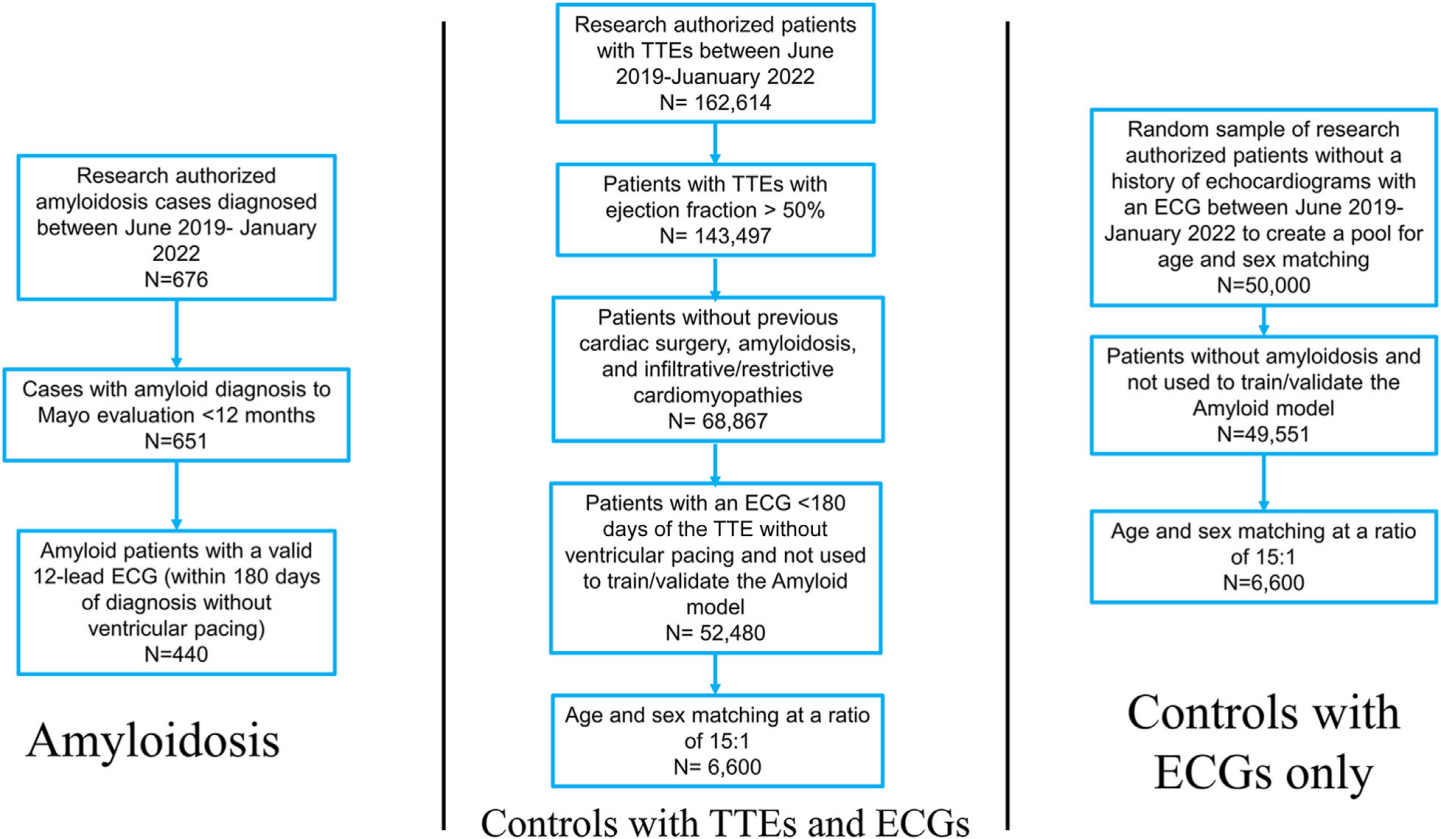
**Patient Flow Diagram** Amyloidosis patients were identified in clinical practice. Those with valid ECGs within 12 months of their initial Mayo Clinic evaluation were evaluated. Of these, patients with ECGs within 180 days of amyloid diagnosis, without ventricular pacing, were included in the final disease cohort. Controls were identified from patients with TTEs during the study time period. Those who met the inclusion and exclusion criteria had TTEs paired with nearest ECG. This group was subsequently matched for age and sex at a disease to control ratio of 1:15. ECG = electrocardiogram; TTE = transthoracic echocardiogram.

The institutional review boards of the Mayo Clinic Foundation approved this study and protocol. Patient consent was waived given the retrospective, minimal-risk nature of this study. Research-authorized patients who were evaluated at the Mayo Clinic for a new amyloid diagnosis (light-chain amyloid [AL] or transthyretin amyloidosis [ATTR]) were identified in the years following algorithm development (n = 676; June 2019-January 2022) from a prospectively maintained institutional amyloid database (Figure 1). Patient age and self-reported race and ethnicity were collected from the Mayo Clinic’s unified data platform. The ECG date, acquisition location, and rhythm were extracted from the MUSE system (GE Healthcare).

For all ECGs, the final rhythm and other ECG findings were adjudicated by a technologist under cardiology supervision. Eligible ECGs were digital, standard 10 second 12-lead ECGs acquired in the supine position during the study timeframe.

Patients with a formal diagnosis of AL or ATTR amyloidosis were included using criteria as previously described in our original study (diagnosis confirmation with biopsy [cardiac or noncardiac] for AL and biopsy or positive technetium Tc 99m pyrophosphate scintigraphy for ATTR; date of biopsy or imaging diagnosis of amyloid defined the date of amyloid diagnosis, even if outside of the Mayo Clinic) including the requirement that patients exhibit cardiac involvement of disease with one of the following: troponin T >0.03 ng/L, N-terminal pro-B-type natriuretic peptide >332 pg/mL, intraventricular septal thickness >12 mm on transthoracic echocardiogram (TTE), or other cardiac symptoms not attributable to other underlying cardiac conditions.^10^ Patients were excluded if they received an amyloidosis diagnosis >12 months before or after their initial Mayo Clinic evaluation (n = 651). Eligible amyloid patients were included in the final cohort if they had a 12-lead ECG during the study timeframe at our institution within 180 days without ventricular pacing at the time of ECG collection (n = 440) (Figure 1). In the case of multiple ECGs, the nearest ECG to amyloid diagnosis was selected.

Potential controls were identified from all research authorized adult patients who either had TTE between June 2019 and January 2022 (n = 162,614) or just ECGs without ventricular pacing between June 2019 and January 2022. All TTEs had a full report coded by a supervising cardiologist. Patients were excluded if the ejection fraction was <50% (n = 143,497). Patients with TTEs were screened for the absence of previous cardiac surgery, amyloidosis, and infiltrative/restrictive cardiomyopathies. Successfully screened patients (n = 68,867) not involved in the original amyloid study then needed to have a 12-lead ECG recording within 180 days of the TTE without ventricular pacing at the time of ECG collection. In the case of multiple ECGs and echoes per patient, the nearest ECG to a TTE was selected to form a control patient pool of 52,480 patients and 55,393 ECG-Echo pairs for age and sex matching. For the control pool without a history of echocardiograms, a random sample (N = 50,000) of research-authorized patients were used to create a pool of 49,551 patients for age and sex matching after excluding patients used to train the 2019 algorithm and any cases. Except when mentioned, all results reported use the control group with ECG-TTE pairs.

Using the confirmed and validated amyloid cases (n = 440), controls were age and sex matched to the disease cohort at a ratio of 15:1 (n = 6,600) (Figure 1) to better mimic the lower prevalence of disease in the general population in contrast to the derivation study (disease-to-control 1:1 ratio).^10^ Similar disease-to-control ratios have been utilized in our other AI-ECG derivation and validation studies for other cardiovascular pathologies.^11^, ^12^, ^13^

All patients included in this study were seen and evaluated at the Mayo Clinic. While this is an internal validation effort, as the original AI-ECG algorithm was developed from the same patient demographic/population, none of the patients in these analyses participated in the derivation of algorithm, and all ECGs in the analysis were acquired after the model was fully developed.

### Artificial intelligence model

The AI model used in this study has been previously described.^10^ Briefly, 3 data sets—training (n = 2,997; 60%), validation (n = 999; 20%), and testing (n = 999; 20%)—had been chosen to ensure an adequate number of cases in the subsets, and each patient was uniquely assigned to a single group. The model architecture was identical to that of a network published previously by our group for predicting the patient’s sex from the ECG,^17^ with inputs consisting of raw ECG waveform data and a binary output predicting the presence or absence of cardiac amyloid. We emphasize that only raw data were used; no manually extracted ECG features were used.

Training consisted of runs of 30 epochs with a batch size of 32 and a learning rate of 0.001. We used a categorical cross-entropy loss on one-hot encoded labels of ATTR or AL amyloidosis as the positive case and no-amyloid as the negative case. Our optimizer was Adam, and the final layer activation was softmax.^18^ Alternative combinations of batch size and learning rate were systematically assessed using the validation set with the ones reported here having the highest performance. The optimal threshold (0.485) was selected utilizing the Youden’s index in this original study.^10^^,^^19^ The same Youden’s index-selected threshold (0.485) was applied in this present study.

Although the model was developed and tested prior to the start date of the current study, to avoid any data leakage, patients used to develop the model were excluded from the current analysis, as we assume that the model might have an unfair advantage when testing the same patients again due to the biometric information in the ECG.

### Main outcome

Standard receiver operator characteristics (ROCs) were calculated including sensitivity, specificity, and AUC curve with corresponding 95% CIs for the detection of amyloidosis. AUC CI calculation was based on the Wald standard error estimate, while exact binomial confidence limits were calculated for sensitivity and specificity. This was done with respect to multiple factors: time (in 1-month blocks, for each month from June 2019 to January 2022), patient age (in 10-year intervals), patient sex, race and ethnicity, amyloid subtype, ECG recording location, and ECG features at the time of screening. Diagnostic odds ratios for the AI-ECG algorithm were performed for each of these measures defined as the ratio between the odds of test positivity in a patient with disease and the odds of test positivity in a patient without disease.

The focus of the statistical analysis was to provide descriptive data on the performance of the algorithm over the range of patient profiles identified above. No formal hypothesis testing was performed to test for differences from the original AUC. Data were presented in forest plots that also provided an estimate of sensitivity, specificity, positive predictive value, and negative predictive value. Data analysis was conducted using Python version 3.9.7.

## Results

**Figure 2:**
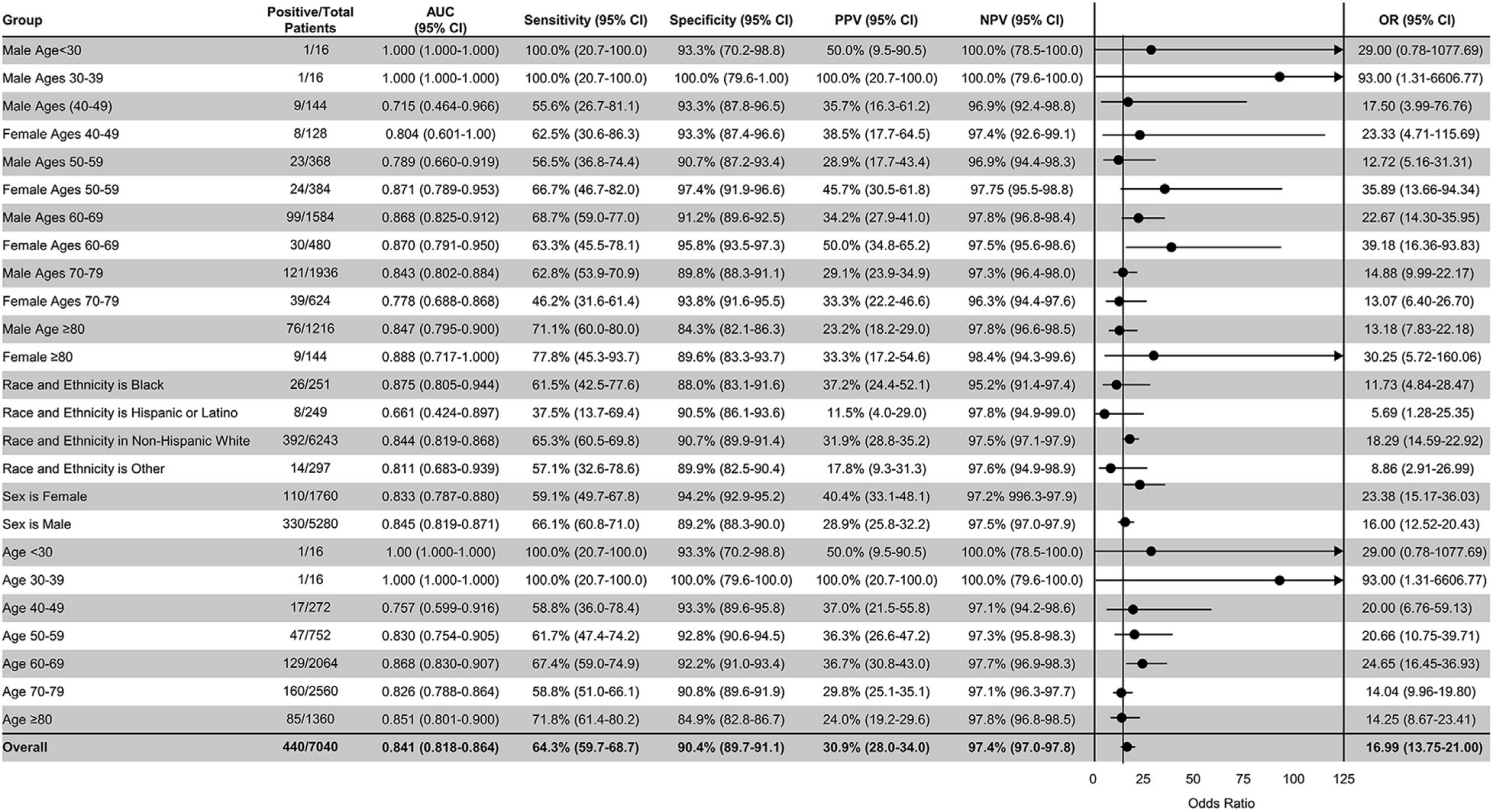
**Performance Analysis of the AI-ECG for Amyloidosis With Respect to Multiple Demographic Characteristics** Area under the curve, sensitivity, specificity, positive predictive value, negative predictive value, and diagnostic odds ratio are reported with 95% CIs for multiple demographic characteristics. Diagnostic odds ratios are visually demonstrated in the forest plot defined as the ratio between the odds of test positivity in a patient with disease and the odds of test positivity in a patient without disease. AI = artificial intelligence; AUC = area under the curve; ECG = electrocardiogram; n = number of amyloid patients in a given subgroup; N = number of study subjects; NPV = negative predictive value; PPV = positive predictive value.

**Figure 3:**
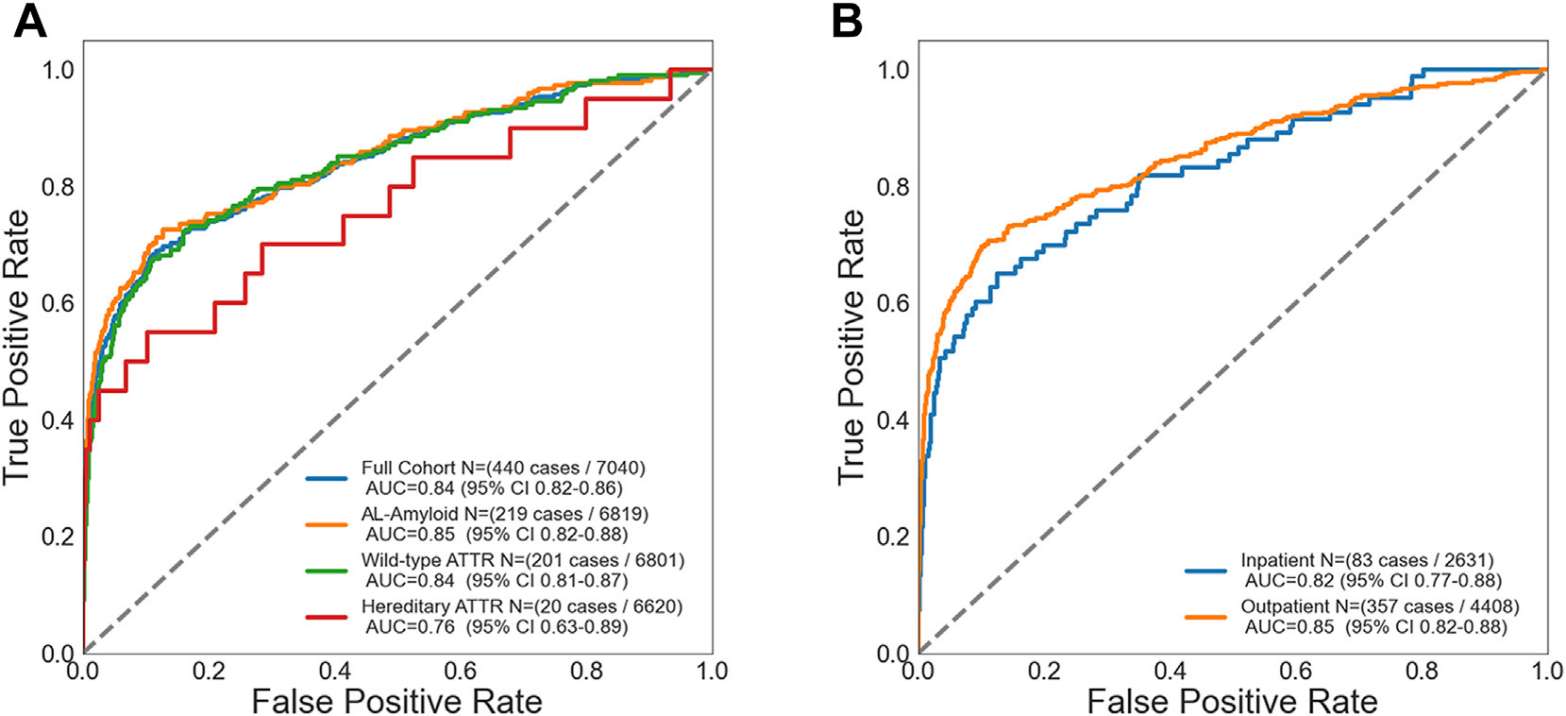
Algorithm Performance With Respect to Amyloidosis Subtype and ECG Recording Location (A) AI-ECG for amyloidosis performance with respect to amyloid subtype and (B) ECG recording location. Graphic demonstration of the receiver operating characteristic for the AI-ECG algorithm to identify amyloidosis among the (A) various amyloid subtypes: AL (N = 219), wild-type ATTR (N = 201), and hereditary ATTR (n = 20) cardiac amyloidosis. The graph on the right (B) demonstrates AI-ECG algorithm performance using inpatient or outpatient ECGs. One ECG in the location subgroup was unable to have a verified location of recording. AI = artificial intelligence; AL = light chain amyloidosis; ATTR = transthyretin amyloidosis; ECG = electrocardiogram.

The final amyloid cohort included 440 patients with 6,600 sex- and age-matched control subjects at a 1:15 ratio (Figure 1, Table 1). The average age of the overall cohort was 70.4 ± 10.3 years; 25.0% were female, and most patients were White (91.3%). In this validation, the AI-ECG for amyloidosis had an AUC of 0.84 (95% CI: 0.82-0.86) for the overall cohort (Figure 2). At the previously defined diagnostic threshold, the algorithm exhibited robust specificity of 90.4% (sensitivity 64.3%) (Figure 2). There was minimal difference in algorithm performance between AL (AUC 0.85, 95% CI: 0.82-0.88, N = 219) and wild-type ATTR (AUC 0.84, 95% CI: 0.81-0.87, N = 201) amyloidosis. There was an apparent drop in performance for hereditary ATTR (AUC 0.76), but with few cases (N = 20), the confidence interval was wider (95% CI: 0.63-0.89) (Figure 3). There was only a small performance difference between inpatient (AUC 0.82, 95% CI: 0.77-0.88) and outpatient (AUC 0.85, 95% CI: 0.82-0.88) recorded ECGs (Figure 3). We finally evaluated whether age and sex matching controls without echocardiograms would impact algorithm performance (control group of ECG-only patients). The AUCs and confidence intervals for the overall cohort and for each amyloid subtype were similar to the echocardiogram control cohort (overall AUC 0.85) (Supplemental Figure 1).

**Table 1:** Baseline Cohort Characteristics

	AL-Amyloid	ATTR-wt	ATTR-v	Total Amyloid Cases	Control
Patients	2.96 (219)	2.72 (201)	0.27 (20)	5.96 (440)	89.18 (6,600)
Age, y	66.50 ± 9.72	75.67 ± 8.07	60.95 ± 11.47	70.44 ± 10.32	70.44 ± 10.31
Male	59.81 (131)	93.03 (187)	60.00 (12)	75.00 (330)	75.00 (4,950)
Race and ethnicity					
Non-Hispanic White	90.41 (198)	93.03 (187)	35.00 (7)	89.09 (392)	88.65 (5,851)
Black	3.65 (8)	4.48 (9)	45.00 (9)	5.91 (26)	3.41 (225)
Other	4.57 (10)	0.50 (1)	15.00 (3)	3.18 (14)	4.29 (283)
Hispanic	1.37 (3)	1.99 (4)	5.00 (1)	1.82 (8)	3.65 (241)
Electrocardiogram					
AFL/AF	14.61 (19)	32.84 (66)	5.00 (1)	19.54 (86)	11.42 (754)
First degree AV block	27.40 (45)	25.37 (51)	20.00 (4)	22.73 (100)	12.64 (834)
Second degree AV block	1.37 (3)	7.46 (15)	5.00 (1)	4.32 (19)	1.46 (96)
Infarction	26.94 (59)	28.36 (57)	35.00 (7)	27.73 (123)	15.02 (991)
Left BBB	0.91 (2)	4.98 (10)	0.00 (0)	2.73 (12)	2.14 (141)
LVH	1.86 (4)	3.48 (7)	5.00 (1)	2.72 (12)	2.70 (178)
Normal sinus	89.04 (195)	65.67 (132)	95.00 (19)	78.64 (346)	87.03 (5,744)
Long QT	17.35 (38)	13.43 (27)	10.00 (2)	15.23 (67)	3.65 (241)
Right BBB	10.50 (23)	15.92 (32)	0.00 (0)	12.5 (55)	11.77 (777)
Low voltage	22.83 (50)	9.95 (20)	5.00 (1)	16.14 (71)	4.47 (295)

Values are % (n) or mean ± SD. AFL/AF = atrial flutter/atrial fibrillation; AL = light chain amyloidosis; ATTR-v = hereditary transthyretin amyloidosis; ATTR-wt = wild type transthyretin amyloidosis; AV = atrioventricular; BBB = bundle branch block; LVH = left ventricular hypertrophy.

### AI-ECG demographic performance

There were minimal differences in algorithm performance based on sex with an AUC of 0.85 (95% CI: 0.82-0.87) for males and 0.83 (95% CI: 0.79-0.88) for females. When evaluating the algorithm based on age and sex, there was consistent performance with 0.77 to 0.88 AUC across the groups except for 3 outlier groups: male <30 years of age, male 30 to 39 years of age, and male 40 to 49 years of age. For males <30 years of age and males between 30 and 39 years of age, there was only one positive case (Figure 2). The AUC for the 40- to 50-year-old males was lower (AUC 0.72; 95% CI: 0.46-0.97), but the wide confidence intervals overlap with the performance of their older counterparts.

To determine if the model exhibited racial bias, the algorithm was analyzed with respect to self-reported race and ethnicity (Table 1). The AI-ECG algorithm exhibited strong performance in the Black, White non-Hispanic, and “other” race and ethnicity subgroups with AUCs ≥0.81 but decreased performance in the Hispanic or Latino race and ethnicity subgroup with an AUC of 0.66 (95% CI: 0.42-0.90) (Figure 2).

### AI-ECG temporal evaluation

**Figure 4:**
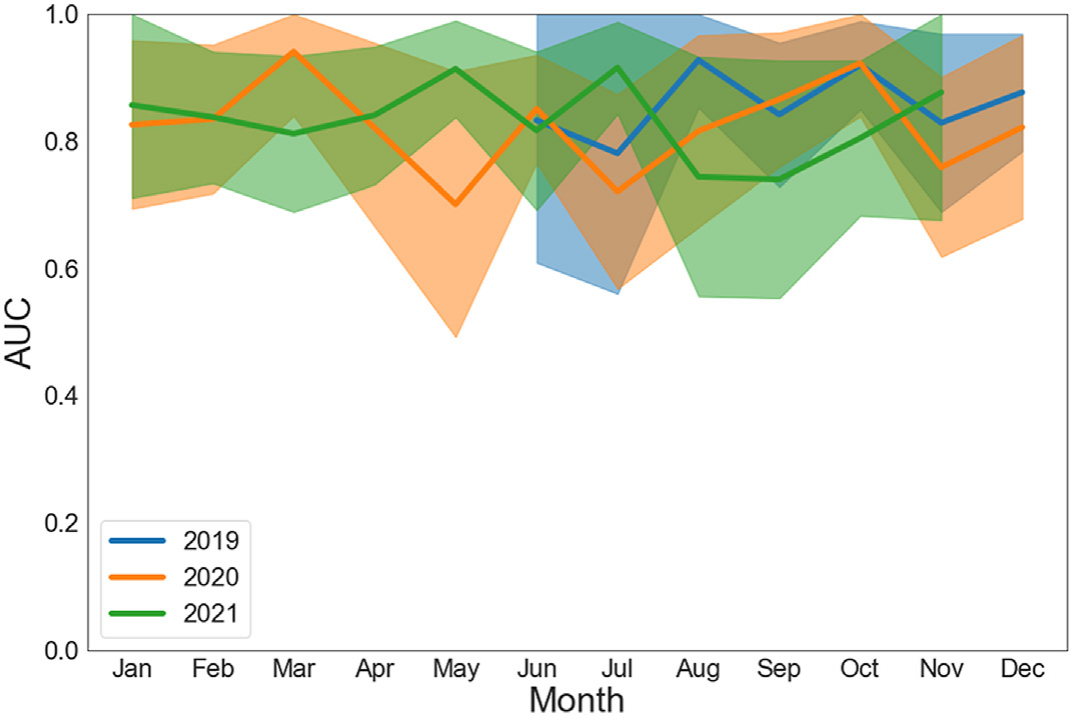
**AI-ECG for Amyloidosis Monthly Time-Series Analysis During Study Timeframe** Month-to-month AUC analysis with 95% confidence intervals. This analysis excluded April 2020, December 2021, and January 2022 within the study timeframe as there was notable bias due to very low number (n = 0-4) of amyloid cases resulting in skewed results. AI = artificial intelligence; AUC = area under the curve; ECG = electrocardiogram.

A time-series analysis of the algorithm is shown in Figure 4. This analysis excluded April 2020, December 2021, and January 2022 within the study timeframe as there were skewed results due to a very low number of amyloid cases (n = 1-3). While there is month-to-month variability in performance, the AUC never dropped below 0.70 with overlapping 95% confidence intervals. Mean annual performance was similar between the years evaluated (AUC 0.82-0.86 for 2019-2021), and there was no consistent data shift of the algorithm over time. There was no significant proportional difference between inpatient and outpatient recorded ECGs (Supplemental Table 1).

### ECG characteristic evaluation

**Figure 5:**
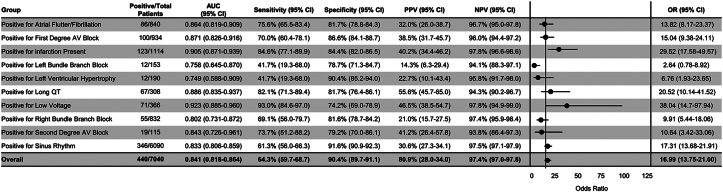
**AI-ECG for Amyloidosis Performance in the Setting of Various Arrhythmias and ECG Features** Area under the curve, sensitivity, specificity, positive predictive value, negative predictive value, and diagnostic odds ratio are reported with 95% CIs for each multiple demographic characteristics. Odds ratio is visually demonstrated in a forest plot. Odds ratios are ‘diagnostic odds ratio’ defined as the ratio between the odds of test positivity in a patient with disease and the odds of test positivity in a patient without disease. AI = artificial intelligence; AUC = area under the curve; AV = atrioventricular; ECG = electrocardiogram; LVH = left ventricular hypertrophy; N = number of study subjects; NPV = negative predictive value; PPV = positive predictive value.

The AI algorithm was evaluated in the setting of various ECG features (Figure 5). ECG features/findings were evaluated on an individual basis, as some ECG characteristics are mutually exclusive (sinus rhythm and atrial flutter, for example). Of the arrhythmias and ECG features evaluated, the algorithm exhibited the highest performing AUC in patients with “low voltage” ECGs (AUC 0.92), followed by any type of infarct pattern (ie, including anterior, septal, lateral, and inferior; AUC 0.90). Patients with left ventricular hypertrophy (AUC 0.75) and left bundle branch block (AUC 0.76) demonstrated the weakest algorithmic performances. Patients with sinus rhythm ECGs exhibited algorithm performance similar to the overall cohort (AUC 0.83; 95% CI: 0.81-0.86) (Figure 5).

## Discussion

In this study, we analyzed the postdevelopment performance of the AI-ECG algorithm for the detection of cardiac amyloidosis with respect to multiple potential confounding factors. This internal validation effort confirmed the algorithm’s robust overall performance regardless of amyloid subtype (AL vs ATTR [wild-type]), ECG recording location, or time since algorithm development. We further demonstrated that the AI algorithm maintained acceptable performance between all age groups, between sexes, and among White, Black, and patients of “other” races with a less robust performance in the smaller Hispanic subpopulation. Finally, we demonstrated the algorithm’s strong performance with respect to multiple ECG features with the strongest performance in ECGs with low voltage and infarct patterns. The AI-ECG performance was less strong in ECGs with left bundle branch block (LBBB) and left ventricular hypertrophy (LVH) (Central Illustration).

### AI-ECG validation and comparison to similar algorithms

It was noted that the AUC for the overall algorithm performance (AUC 0.841) in this validation study is lower than the original derivation work (AUC 0.91; 95% CI: 0.90-0.93).^10^

There are a few hypotheses for this difference. In this validation study, our amyloid population was 55.7% ATTR cardiac amyloid compared to 21% ATTR in the derivation study. This ATTR prevalence difference in the original cohort is likely a reflection of the amyloid patient population from 2000 to 2019, with the development of nuclear imaging techniques to detect and diagnose ATTR during the latter half of this time period (ie, technetium pyrophosphate imaging).^20^ As such, the algorithm may be able to identify AL cases with cardiac involvement better than ATTR as a result of this original training environment, consistent with our subanalysis results (Figure 3). It is also worth noting that patients with ATTR were included on the basis of tissue diagnosis *or* positive pyrophosphate scintigraphy. As such, patients with a negative pyrophosphate imaging study may not have undergone cardiac biopsy and have the potential to be erroneously included in the control of our study in the absence of classic amyloid findings on echocardiogram, and vice versa (ie, while rare, patients with a positive imaging study may not have cardiac ATTR but were included in the disease cohort). Another consideration is the postdevelopment “Covid-era,” where there were many patients at the Mayo Clinic receiving ECGs and TTEs that were “sicker” than the general population, which may have triggered false-positive or skewed AI-ECG results in the control group. However, our supplemental analysis with an ECG-only control group (ie, control patients without TTE on record) suggested this hypothesis was unlikely (Supplemental Figure 1). Finally, while amyloid patients in our study “met criteria” for cardiac amyloid, defining the presence or absence of cardiac involvement of this disease entity remains challenging (ie, confounding symptomology, biomarker abnormalities nonspecific to amyloid manifestation). As a result, some patients included in our final disease cohort, even after the application of strict inclusion and exclusion criteria, may not ultimately have cardiac amyloid, though we anticipate this percentage is rather small.

We compared our work to other efforts focused on the detection of cardiac amyloidosis using a variety of AI and machine learning methodologies. Goto et al developed a convolutional neural network for both ECGs and TTEs, which exhibited similarly robust performance to our studies (AI-ECG AUC 0.85-0.91; AI-TTE 0.89-1.00).^21^ This group also demonstrated how a step-wise approach with AI-ECG screening could enhance the positive predictive value of subsequent AI-TTE screening in patients who met AI-ECG-based “high-likelihood” criteria. Interestingly, this group similarly observed a decrease in performance when validating the AI-ECG model at different institutions/environments, while remaining robust in general (AUC >0.85), similar to our study.^21^ It is also worth noting that each of these validation efforts had populations with a higher amyloidosis prevalence compared to our present study with control-disease ratios of 1:1 and 3:1. In a separate machine-learning effort, Huda et al. were able to identify “at-risk” patients with amyloidosis by utilizing Random Forest classification.^22^ This ML algorithm was able to identify wild-type ATTR patients from a small 1:1 cohort with excellent performance (AUC 0.95); however, when attempting validation testing for unspecified “cardiac amyloidosis,” the algorithm exhibited a fair drop in performance (AUC 0.76-0.80).^22^ In each of these efforts, it must be noted that variations in the amyloid population tested, the location of ECG recording, and potentially the timing of ECG recording may have played a role in these performance variations, similar to what was observed in our validation study.

While this small decrease in performance was observed, the strong overall performance of the algorithm is an encouragement that this AI-ECG algorithm for cardiac amyloidosis exhibits acceptable performance in this secondary evaluation, despite multiple potential confounders, similar to other routinely used medical screening tests (ie, mammography, pap smear, natriuretic peptides; AUC 0.67-0.84).^23^, ^24^, ^25^

### AI-ECG performance by demographic characteristics

The AI-ECG algorithm for amyloidosis demonstrated acceptable performance across all patient age groups. As amyloidosis typically impacts patients of increased age, the algorithm shows great promise as a screening tool, particularly demonstrating its strongest and most consistent performance in patients between 50 and 70 years of age. It is also worth noting that there was no statistically notable difference in algorithm performance between male and female counterparts, demonstrating that our algorithm is essentially absent of sex bias.

Similar to age- and sex-subanalyses, the AI-ECG for amyloidosis also exhibited acceptable performance for both White, Black, and patients of “other” racial backgrounds (ie, Asian, Pacific Islander, and so on; AUC >0.81 for all), while patients of Hispanic ethnicity exhibited a decrease in AUC (0.66), with wide confidence intervals (AUC, 95% CI: 0.42-0.90) likely representative of the small sample size tested (n = 8 amyloid; total cohort, N = 241). Though a race-specific subanalysis was not reported in the initial AI-ECG amyloid study, this original cohort was 93.4% White, similar to our present study.^10^ As a result, the noted variability in algorithm performance among Hispanic patients may also be a representation of the algorithm’s initial training environment. While this performance variability between race/ethnic populations does not currently exhibit a statistically significant limitation of the algorithm, AI-ECG results in these underrepresented groups must be interpreted with caution within appropriate clinical context, particularly as future external validation efforts may further clarify the algorithm’s validity in these subpopulations. While one may consider this algorithm as a potential screening tool for patients of African descent with increased prevalence of the gene responsible for heritable ATTR amyloidosis,^26^ the algorithm’s strong performance in this Black subpopulation (AUC 0.88) may be confounded by the algorithm’s lesser performance for patients with heritable ATTR amyloidosis (AUC 0.76). This potential use would require a specific investigative effort to assess the validity in this special population.

### AI-ECG time series and location analysis

When evaluating the algorithm over time, there was a slight performance deviation in May 2020, during the initial surge of the COVID-19 pandemic across the United States. As our algorithm currently maintains a “black box” model, it is difficult make conclusions as to what specifically during this period impacted the algorithm. It was hypothesized there were likely a higher ratio of ECGs recorded in the inpatient/hospital setting with more confounding variables present impacting algorithm performance (ie, acute illness, invasive procedures, new medications impacting the cardiovascular system), which we have noted in a similar AI-ECG study.^15^ However, we did not find that consistent correlation in this present study, as the proportional amount of inpatient/outpatient ECGs (control and disease) were similar month-to-month including May 2020 (Supplemental Table 1). We do find reassurance that the mean annual performance was nearly unchanging (AUC 0.82-0.86) without consistent shift in model performance over time with overlapping 95% confidence intervals on a month-to-month basis. Further study 5 and 10 years after model development will further determine if recalibration or retraining over time is required for this AI-ECG algorithm.

### AI-ECG performance and ECG features

Traditionally, the typical ECG features representative of cardiac AL amyloidosis are low QRS voltage (<0.5 mV) or pseudoinfarct pattern (Q-wave in 2 contiguous leads without presence of coronary disease).^27^^,^^28^ We noted the AI-ECG for amyloid exhibited its best performance in each of these ECG subgroups (Figure 5) (low voltage, infarction present). However, an important clarification remains about the AI-ECG performance in these subgroups. While the AI-ECG algorithm may perform best in these ECG subcategories with classically “amyloid” findings, the AI-ECG is not identifying amyloid patients solely as a result of this ECG pattern, as the controls in this subanalysis have the same ECG features. Rather, the AI-ECG is able to see beyond what is visible and distinguish a patient with low ECG voltage who may have this finding as a result of underlying disease apart from a patient with low ECG voltage from a secondary cause (ie, extra subcutaneous tissue; or similarly, pseudo-infarct from amyloid vs true infarct from coronary disease). This finding is actually surprising, as one could imagine this is a more challenging feat for the algorithm to distinguish amyloid from healthy patients with similar ECG features. Understanding what precisely allows the AI algorithm to make these diagnostic determinations is an area for future study.

In the ECG subgroup analysis, LBBB and LVH demonstrated the lowest sensitivities (both 42%) with LVH demonstrating the lowest overall performance with an AUC of 0.75. These performance deviations are not entirely unexpected given the challenges of traditional ECG interpretation with conduction abnormalities and the stark difference between these ECG findings and “classical” amyloid ECG findings. As a result, use of the AI-ECG amyloid algorithm in patients with these ECG features should be done with caution. However, regardless of ECG characteristics, this algorithm performs at a level comparable to many commonly used, previously mentioned medical diagnostic tests (AUC 0.67-0.84^23^, ^24^, ^25^).

As a whole, this study demonstrates the robust performance of this AI-ECG screening tool among a variety of ECG features that could help appropriately elevate clinical suspicion for potential amyloid in patients undergoing cardiology evaluation.

### Study Limitations

There are several limitations to our study. The cases included patients with AL and ATTR amyloidosis with various manifestations of cardiac infiltration, recognizing that determination of cardiac involvement is challenging in some patients. However, cardiac amyloidosis is underdiagnosed, even potentially at the Mayo Clinic, and there may have been patients in the controls with “undiagnosed,” subtle disease. While the performance of the algorithm was acceptable between amyloid subtypes, it is worth noting that the hereditary amyloid cohort is quite small (n = 20) resulting in a fair performance drop compared to the nonheritable subtypes, suggesting a need for further validation in this subpopulation. On a similar note, the overall population lacked racial/ethnic diversity. While there was statistically acceptable algorithm performance between racial and ethnic subgroups, conclusions are limited due to small sample size, particularly as the Hispanic subgroup represented in our study exhibited lower performance and wide confidence intervals. We urge providers using such AI-based ECG technology to interpret results with caution in the appropriate clinical context, given this potential limitation. It is finally worth noting that there are some potential limitations in algorithm performance with respect to certain ECG features, namely, LVH and LBBB.

This analysis was completed at a single institution in a single geographic location, and the results from this study may not be widely applicable. Further prospective validation efforts at variable locations and institutions would allow for a more comprehensive assessment of the algorithm’s performance.

## Conclusions

In this study, we demonstrated the stability of the AI-ECG algorithm for amyloidosis with respect to patient age, sex, race, recording location, multiple ECG features, and over time. We did observe fluctuations of algorithm performance in the Hispanic subpopulation and in patients with certain ECG features (ie, LBBB and LVH), which carry implications for application of this algorithm in the clinical setting.

## Funding support and author disclosures

This work was funded by the 10.13039/100000871Mayo Clinic Department of Cardiovascular Medicine and the Mayo Cardiovascular Research Center with no industry support. This research did not receive any specific grant from funding agencies in the public, commercial, or not-for-profit sectors. Dr Harmon has received support from the NIH StARR Resident Investigator Award (NIH 5R38HL150086-02). Dr Grogan has received research support from Alnylam, Eidos, Janssen, Pfizer, and Prothena; and consulting and/or advisory board compensation (paid to Mayo Clinic, no personal compensation) from Akcea, Alnylam, Eidos, Pfizer, Prothena, and Janssen. Dr Dispenzieri has received research support from Celgene, Takeda, Janssen, Pfizer, and Alnylam; and is on the advisory board for Janssen. Drs Grogan, Lopez-Jimenez, Dispenzieri, Attia, Friedman, and Murphree and Mayo Clinic have licensed the algorithm described in this work to Anumana and may benefit from its commercialization. All other authors have reported that they have no relationships relevant to the contents of this paper to disclose.

PERSPECTIVES **COMPETENCY IN MEDICAL KNOWLEDGE:** This validation study demonstrates the strong performance of the AI-ECG algorithm postdevelopment to detect cardiac amyloidosis with respect to multiple potential confounders (ie, age, sex, race, amyloid subtype, recording location, and ECG features) with promising application for clinical practice.

**TRANSLATIONAL OUTLOOK:** The robust AI-ECG algorithm could be an impactful clinical screening tool for both light-chain and ATTR. Future prospective clinical study should focus on the application and integration of this AI-ECG algorithm into clinical practice to better understand its utility as a screening tool for this frequently underrecognized pathology.
