# Supplementary material for: Postdevelopment Performance and Validation of the Artificial Intelligence-Enhanced Electrocardiogram for Detection of Cardiac Amyloidosis
Source: JACC Adv. Author manuscript; Available in PMC 2024 Apr 18. (PMC11025724; doi:10.1016/j.jacadv.2023.100612)
Supplement: supplement [file NIHMS1968741-supplement-supplement.pdf]

Supplemental Figure 1: AI-ECG for amyloidosis performance with respect to amyloidosis subtype using controls without echocardiograms

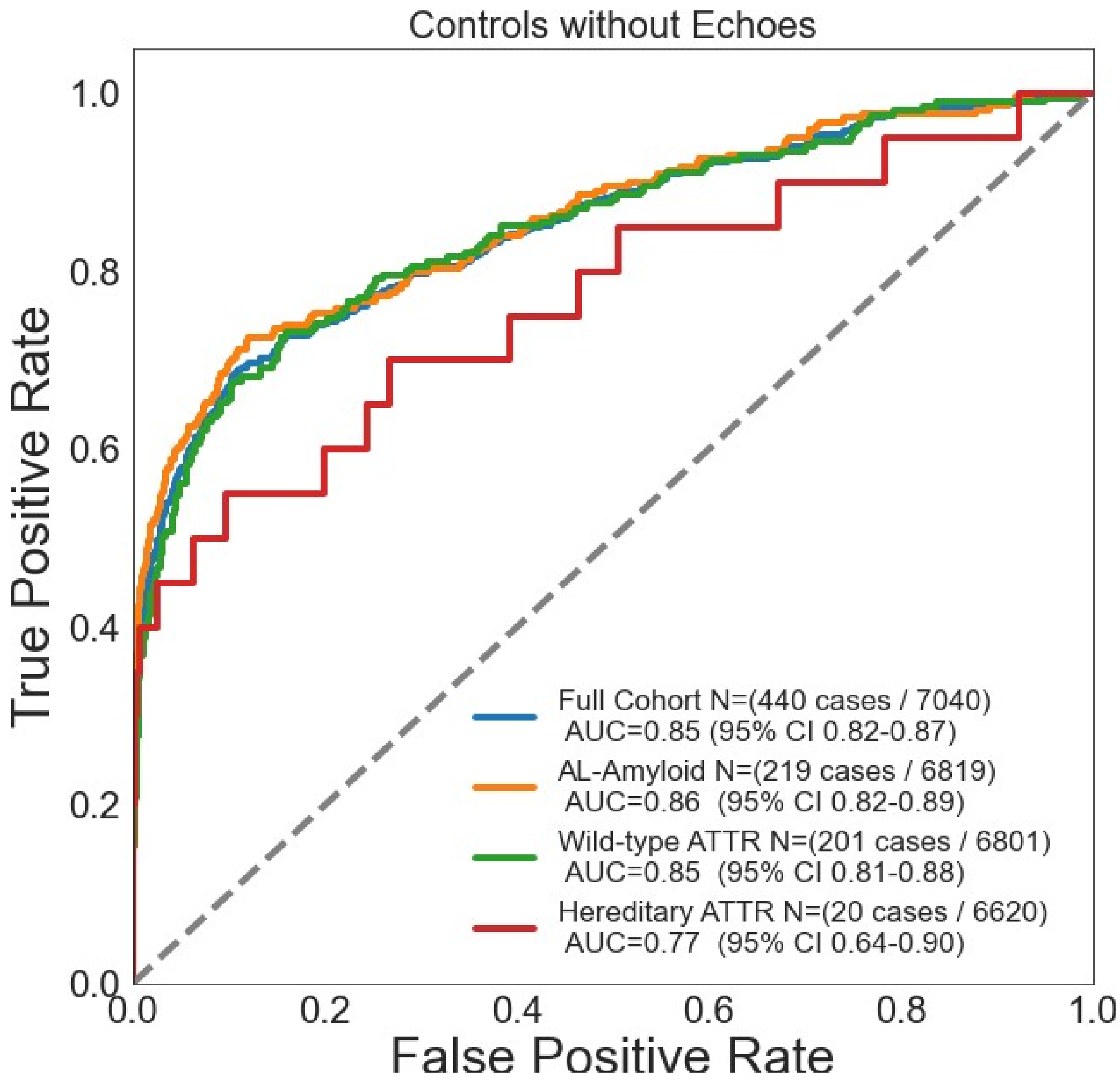

Supplemental Table 1: Inpatient vs Outpatient recorded ECGs. April 2020, December 2021, and January 2022 are excluded as in Figure 4.

ECG Month Year	Inpatient	Outpatient	% Inpatient
6/1/19	59	112	34.50
7/1/19	72	101	41.62
8/1/19	78	128	37.86
9/1/19	77	135	36.32
10/1/19	57	139	29.08
11/1/19	66	119	35.68
12/1/19	67	92	42.14
1/1/20	77	147	34.38
2/1/20	67	142	32.06
3/1/20	61	83	42.36
5/1/20	47	108	30.32
6/1/20	83	129	39.15
7/1/20	68	132	34.00
8/1/20	93	145	39.08
9/1/20	81	164	33.06
10/1/20	77	156	33.05
11/1/20	81	116	41.12
12/1/20	78	117	40.00
1/1/21	91	141	39.22
2/1/21	86	153	35.98
3/1/21	88	194	31.21
4/1/21	97	222	30.41
5/1/21	98	169	36.70
6/1/21	102	172	37.23
7/1/21	100	140	41.67

**8/1/21**	**101**	**156**	**39.30**
**9/1/21**	**100**	**161**	**38.31**
**10/1/21**	**108**	**169**	**38.99**
**11/1/21**	**99**	**138**	**41.77**
